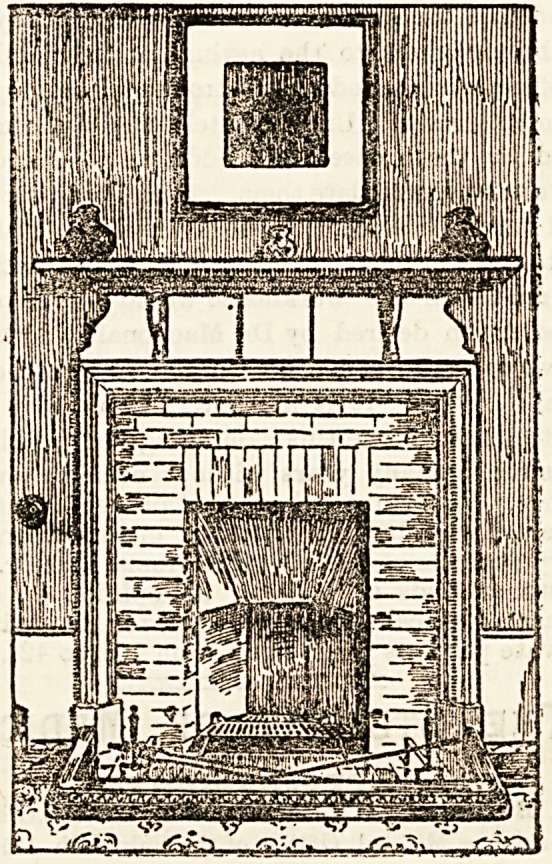# Practical Departments

**Published:** 1904-04-23

**Authors:** 


### PRACTICAL DEPARTMENTS.

### HEATING.

We illustrate this week "The Rational" fireplace, manufactured by Messrs. Joshua W. Taylor, Limited, which contains some interesting details.

The main points in this fireplace are as follows:—(1) A fire on a hearth level with the floor, and yet there is a dustpan beneath it which is easily removable while the fire is burning. (2) The heat from the fire readily radiates towards the feet. (3) The floor can be swept up towards the fireplace, and this does away with that abominable nuisance in a sick-room, *i.e.* the clattering dustpan, a point which alone should recommend it to those in charge of our suffering fraternity.

If we turn to the section we shall at once be struck with the absence of any air-duct beneath the fire, and yet, as we saw upon the occasion of our visit and can therefore testify, it burns well without any special air-feed, and this fact impressed itself upon us when we tried to remove the dustpan by the projecting handles without any stove gloves, suggesting an improvement which might be effected by lengthening them. But this is a detail.

To start the fire the front grid, which is hinged for the purpose, is folded up into a vertical position in which it remains until the fire is well alight, when it is pushed down flat and is then in its proper place and no time need be wasted upon cleaning and blacking bars, etc.

A capital sheet of drawings to one-quarter full size and fully annotated is given with each fireplace sold, and upon this there is a special note of warning to the effect that, owing to the intense heat thrown out, no woodwork should be placed nearer the fire than 9 inches, and that in old buildings the hearth should always be taken up, and timber construction should be searched for. This, however, is not a greater restriction than that which is wisely imposed upon architects and others by the London Building Act of 1894, which provides that "No woodwork shall be placed nearer than 12 inches to the face of a flue."

We have said much that is in favour of this novel little fireplace, and it would be wonderful indeed if there were no drawbacks to its use, for here it should be pointed out that when fitting one of these fireplaces to an old building we may be brought face to face with a little difficulty upon the upper floors owing to that other clause in the Building Act which states that "Timber or woodwork shall not be placed under any chimney opening within 10 inches from the upper surface of the hearth," which in this case would mean the upper surface of the cast-iron bottom plate, and in consequence it might necessitate the provision of a raised hearth. In the drawing supplied a simple groove is shown for the support of the grid, but this is liable to get filled up with ashes or to get chipped, and the makers have now wisely added feet to their grids, so there need be no further apprehension upon this score.

At first sight the holes in the grids appeared to us to be small, but this is accounted for by the slow combustion action which slowly eats up the fuel in such a way that it leaves only a fine dust, and it is stated by the makers that besides burning throughout a night without attention, the "Rational" will consume coal, peat, or wood economically.

It is undoubtedly a capital fireplace, and an inspection of the wares in the showrooms at No. 135 Victoria Street, Westminster, will be both edifying and instructive.

The designs of many of the mantels have been kept well in hand, and for the most part depict a quiet reserve dear to the heart of the artistic.

## Figures and Tables

**Figure f1:**
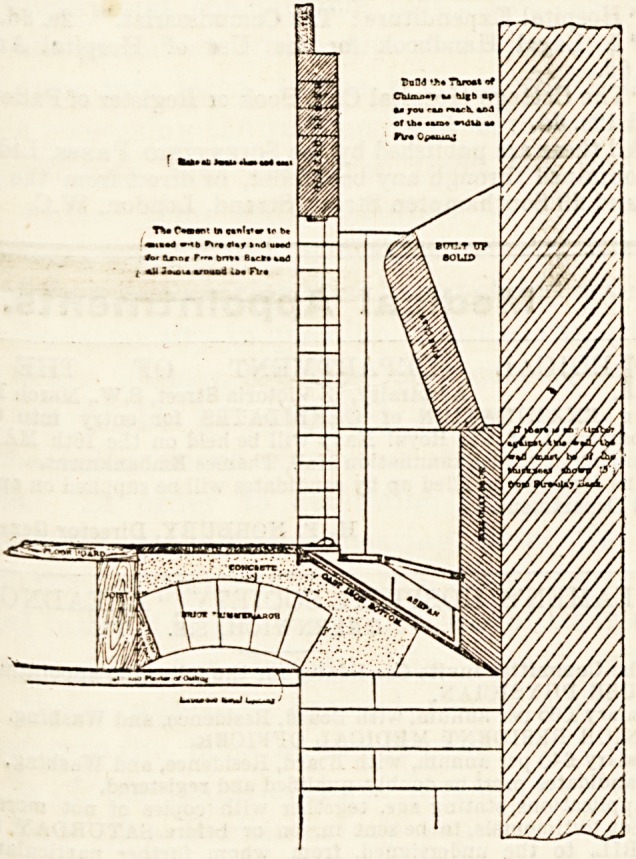


**Figure f2:**